# Bioinspired Photo-Responsive Liquid Gating Membrane

**DOI:** 10.3390/biomimetics7020047

**Published:** 2022-04-18

**Authors:** Rongrong Zhang, Jinmei Lei, Jiadai Xu, Hexuan Fu, Yuan Jing, Baiyi Chen, Xu Hou

**Affiliations:** 1 State Key Laboratory of Physical Chemistry of Solid Surfaces, College of Chemistry and Chemical Engineering, Xiamen University, Xiamen 361005, China; zhangrr@stu.xmu.edu.cn (R.Z.); leijinmei1021@xmu.edu.cn (J.L.); xujiadai@stu.xmu.edu.cn (J.X.); 20420182201929@xmu.edu.cn (H.F.); 2 Department of Physics, Research Institute for Biomimetics and Soft Matter, Fujian Provincial Key Laboratory for Soft Functional Materials Research, Jiujiang Research Institute, College of Physical Science and Technology, Xiamen University, Xiamen 361005, China; 19820201153563@stu.xmu.edu.cn; 3 Innovation Laboratory for Sciences and Technologies of Energy Materials of Fujian Province (IKKEM), Xiamen 361005, China

**Keywords:** stomata-inspired, photo-responsive, liquid gating membrane, gas transport

## Abstract

Stomata in the plant leaves are channels for gas exchange between the plants and the atmosphere. The gas exchange rate can be regulated by adjusting the opening and closing of stoma under the external stimuli, which plays a vital role in plant survival. Under visible light irradiation, the stomata open for gas exchange with the surroundings, while under intense UV light irradiation, the stomata close to prevent the moisture loss of plants from excessive transpiration. Inspired by this stomatal self-protection behavior, we have constructed a bioinspired photo-responsive liquid gating membrane (BPRLGM) through infusing the photo-responsive gating liquid obtained by dissolving the azobenzene-based photo-responsive surfactant molecules (AzoC_8_F_15_) in N,N-Dimethylacetamide (DMAC) into nylon porous substrate, which can reversibly switch the open/closed states under different photo-stimuli. Theoretical analysis and experimental data have demonstrated that the reversible photoisomerization of azobenzene-based surfactant molecules induces a change in surface tension of the photo-responsive gating liquid, which eventually results in the reversible variation of substantial critical pressure for gas through BPRLGM under alternating UV (*P*_Critical_ _(off)_) and visible (*P*_Critical_ _(on)_) light irradiations. Therefore, driven by a pressure difference Δ*P* between *P*_Critical_ _(on)_ and *P*_Critical_ _(off)_, the reversible switches on the open/closed states of this photo-responsive liquid gating membrane can be realized under photo-stimuli. This bioinspired membrane with switchable open/closed liquid gating performance under photo-stimuli has the opportunity to be used in the precise and contactless control of microfluidics.

## 1. Introduction

Many natural living organisms can adjust their behaviors according to changes in their surroundings, which has inspired the design of various bioinspired materials with superior properties [1,2,3,4]. One intriguing example is that guard cells can control the opening or closing of the stomata on a leaf surface in accordance with the variation of environmental conditions, (e.g., light, humidity and CO_2_ concentration), regulating the rate of gas exchange between plants and their surroundings [5,6]. Visible light irradiation can induce stomata to open to ensure the normal life process of plants, but under intense UV light irradiation, stomata will close to prevent excessive moisture loss. This self-protection behavior of plant stomata provides a new strategy for designing smart bioinspired materials with gating performance [7,8]. Inspired by the stomata on the leaf surface that regulate the movement of moisture and gas into and out of leaves, Raghavan et al. designed a smart hydrogel-based valve or membrane, which remains closed to prevent water passing through the valve under ambient conditions, but when triggered by some specific external stimuli, such as temperature, pH or light, allows water to pass through the valve [9]. Han et al. reported a stoma-inspired photomechanical ion channel by coating the azo-based polymers on a poly-carbonate-track-etched membrane. The size of ion channels changes due to the photoisomerization of the azo-based polymer and results in an ionic change in the current value under UV light irradiation, which endows the ion channel with potential applications for rapid and selective light detection [10]. By mimicking the smart opening and closing of stomata, a variety of functional bioinspired materials have been developed and show a wide range of potential applications in the fields of droplet manipulation [11], mass delivery [12] and air filtration [2]. However, previous studies on bioinspired stomatal systems are usually used for smart liquid or ion-transport, based on the deformation of hydrogels [9], and the bioinspired gating systems for smart gas transport are rarely investigated.

In recent years, liquid gating technology was developed by infusing the capillary-stabilized liquid into a porous substrate as a reversible and reconfigurable gate to regulate fluid transport [13,14]. In the liquid gating system, the gating liquid fills and seals the micropores, and transport fluid must deform the gating liquid interface to enter the pores, driven by pressure [15]. In the early stage of liquid gating technology, the continuous and controllable delivery of different fluids was mainly achieved by regulating the system pressure. However, the single pressure-driven mechanism is difficult to meet the complex requirements in practical applications. A variety of external stimulus-responsive liquid gating systems have been developed on the basis of the pressure-driven system. These responsive liquid gating systems will be switched between open and closed states under external stimulus, such as light [16], optothermal [17], CO_2_ [18], electric field and magnetic field [19], and have been successfully applied in the fields of multiphase separation, controllable gas valves and drug delivery [20]. Among these, as a precise controllable and contactless external stimulus, light shows great potential in developing the smart responsive liquid gating system [21,22]. Our previous work reported a light-responsive and corrosion-resistant liquid gating system prepared by incorporating gating liquid and a solid porous membrane grafted with azobenzene-based molecular photoswitches [16]. The substantial critical pressure for gas transport can be regulated by the photoisomerization of azobenzene-based molecular photoswitches under UV light irradiation, which further realized the precise control of gas flow combined with the high spatiotemporal resolution of light. Different from the photochemical mechanism, the photophysical mechanism is usually accompanied by the generation of heat. We also presented a photothermally induced liquid gating system that realized the navigation control of fluid and gas/liquid separation under light stimuli through redistribution of the gating liquid due to the effect of the Marangoni flow [17]. The two aforementioned light induced liquid gating systems are in an open state under external light stimuli. However, due to the intense energy of UV light, it is necessary to initiate the self-protection behavior in many tough application scenarios, such as natural gas transport and chemical reactions involving flammable gas. Therefore, the development of a liquid gating system with a closed state under UV light irradiation is very important.

Inspired by the fluid transport and switchable properties of stomata [6], we have constructed a bioinspired photo-responsive liquid gating membrane (BPRLGM), which can be reversibly switched between closed and open states under alternating UV and visible light irradiations. To realize the photo-responsibility of the liquid gating membrane, we synthesized azobenzene-based photo-responsive surfactant molecules (AzoC_8_F_15_) by a facile one-step substitution method [23,24]. Then, the AzoC_8_F_15_ molecules were dissolved in N,N-Dimethylacetamide (DMAC) to form the photo-responsive gating liquid. The surface tension of the gating liquid can be significantly changed through photoisomerization of AzoC_8_F_15_ molecules under different photo-stimuli, resulting in the corresponding variation of critical pressure for gas through the BPRLGM. Under visible light irradiation, the *trans*-formed AzoC_8_F_15_ molecules closely align horizontally at the gas-liquid interface, leading to a low gating liquid surface tension and low substantial gas transmembrane critical pressure (*P*_Critical (on)_). If the pressure difference on both sides of the membrane (Δ*P*) is higher than *P*_Critical (on)_, gas can pass through the BPRLGM, and the membrane is open. Whereas, under UV light irradiation, AzoC_8_F_15_ molecules undergo a *trans*-to-*cis* photoisomerization, inducing an increase in surface tension of the gating liquid, which increases the critical pressure (*P*_Critical (off)_) for gas through the BPRLGM. If Δ*P* is lower than *P*_Critical (off)_, the BPRLGM will switch to the closed state. Then, the membrane will reopen under visible light irradiation. This BPRLGM, which opened under visible light irradiation and closed under UV light irradiation, will provide many avenues for potential applications [25].

## 2. Materials and Methods

### 2.1. Chemicals

Perfluorooctyl chloride was purchased from Aladdin Industrial Co., Ltd., Shanghai, China. 4-aminoazobenzene was purchased from Macklin Industrial Co., Ltd., Shanghai, China. Tetrahydrofuran (99.5%, Extra Dry) was purchased form Energy Chemical Co., Ltd., Tangshan, China. N,N-Dimethylacetamide and hexane were purchased from Sinopharm Chemical Reagent Co., Ltd., Shanghai, China. Nylon porous membrane (pore size of 8 μm) was purchased from Haining Zhongli Filtering Equipment Factory, Haining, China. Milli-Q water (18.2 MΩ cm) was used in the synthesis experiment. Liquid gating membranes were created by infusing the gating liquid into nylon porous membranes. Air was used as the transport gas during the transmembrane pressure measurements.

### 2.2. Synthesis of Azobenzene-Based Photo-Responsive Surfactant Molecules

AzoC_8_F_15_ was prepared by dissolved 0.1657 g of 4-aminoazobenzene and 0.4356 g perfluorooctyl chloride in 10 mL of Tetrahydrofuran in turn. The mixture was refluxed for 6 h, then the filtrate was collected by filtration. Next, milli-Q water was added to the filtrate to separate the product by filtration, and then purified by recrystallization with hexane. Finally, the product was dried in a vacuum oven overnight.

### 2.3. Surface Tension Measurements

During the surface tension measurements, for both DMAC and photo-responsive gating liquid, the irradiation time for both UV and visible light during alternative irradiation cycles is 15 min to ensure the complete transformation of the photo-responsive surfactant molecules. the volume of liquid droplets is 10 μL, and the value of the surface tension was tested with an average of three independent measurements, at least.

### 2.4. Gas Transmembrane Pressure Measurements

The critical pressure for gas through the liquid gating membrane under UV and visible light irradiations was measured with the self-designed transmembrane pressure measurement device by wet/wet current output differential pressure transmitters (PX273-020DI) from OMEGA Engineering, Inc., (Stamford, CT, USA). The Harvard Apparatus PHD ULTRA Syringe Pump was used to control the gas flow rate with 1 mL/min in all transmembrane pressure measurements.

### 2.5. Characterizations

The photoisomerization of the AzoC_8_F_15_ molecule was measured by UV-visible near-IR spectrophotometer (PerkinElmer, Lambda 1050+, Waltham, MA, USA). Surface tension of AzoC_8_F_15_ solutions was measured by pendant drop (right) on the CA meter (DataPhysics, OCA100, Filderstadt, Germany).

## 3. Results and Discussions

**Figure 1:**
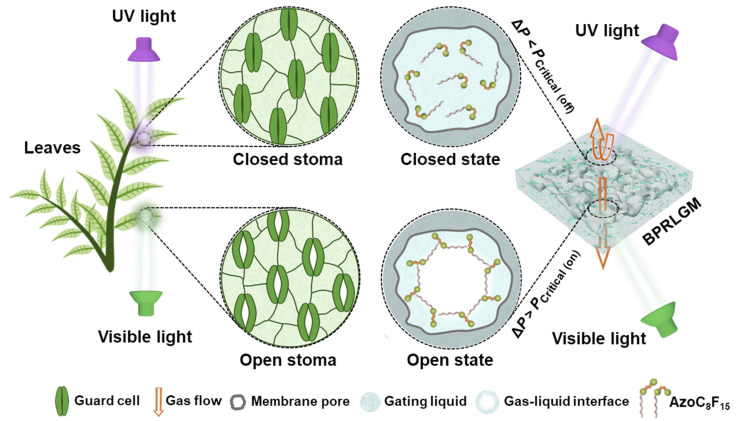
A bioinspired photo-responsive liquid gating membrane (BPRLGM). **Left**: Inspiration from the stoma of leaf. Guard cells will regulate the opening and closing of the stoma in response to changes in external environment. Under visible light irradiation, the stoma will open to perform normal life process, while during midday, transpiration of the plant will be enhanced due to strong UV light irradiation and the stoma will close to prevent water loss. **Right**: Working principle of the bioinspired photo-responsive liquid gating membrane. Schematics of the alignments of photo-responsive surfactant molecules (AzoC_8_F_15_) at the gating liquid surface of BPRLGM under UV and visible light irradiations. The reversible *trans*-to-*cis* photoisomerization of AzoC_8_F_15_ molecules induces the surface tension variation of the photo-responsive gating liquid, which further brings about changes in the substantial critical pressures for gas through BPRLGM.

The stoma, bordered by a pair of guard cells on the surface of a leaf, is the main channel for gaseous exchange between a plant and its surroundings [26]. The gas exchanged through the opening and closing of the stoma consists mainly of carbon dioxide, oxygen and water vapor, which are the raw materials and products of photosynthesis, respiration and transpiration in plants. Guard cells can regulate the opening and closing of the stoma, responding to a change of surroundings, further regulating the rate of gaseous exchange, which plays a critical role in the life process of plants [27]. In general, illumination is the main factor affecting the stomatal behavior: the stoma will open to ensure the normal life processes of plants under mild visible light, and will close to reduce the transpiration rate to avoid the loss of moisture under strong UV irradiation during midday (Figure 1, left). In recent years, human activities have led to the destruction of the ozone layer, resulting in an increase in UV radiation from sunlight. Therefore, the self-protection ability of the stoma in harsh light environments is particularly important for ensuring the normal life processes of plants, which has provided the inspiration for designing various photo-responsive bioinspired systems.

Inspired by the stomatal behavior of plant leaves, we constructed a bioinspired photo-responsive liquid gating membrane (Figure 1, right), in which the closed and open states could be reversibly regulated by alternating UV and visible light irradiations. An azobenzene-based photo-responsive surfactant molecule with a polar photo-responsive azobenzene group and a non-polar fluorocarbon chain is dissolved in DMAC to form the photo-responsive gating liquid. The BPRLGM can be constructed by simply incorporating the gating liquid with a nylon porous membrane. Under alternated UV and visible light irradiations, the AzoC_8_F_15_ molecules undergo reversible *trans*-to-*cis* photoisomerization and the alignment of the AzoC_8_F_15_ molecules at the gating liquid surface changes due to the molecular configuration variation, which further leads to changes in surface tension of gating liquid [28]. According to the Laplace equation:

$$\Delta P=\frac{4\gamma }{d}$$

where γ and d represent the surface tension of the gating liquid and the efficient pore size of the membrane, respectively. Such surface tension variation of the gating liquid further causes the change in the substantial gas transmembrane critical pressures of the BPRLGM under different light irradiations [13].

Under visible light irradiation, the AzoC_8_F_15_ molecules are in *trans*-form and closely align horizontally at the gating liquid surface, which greatly reduces the surface tension of the gating liquid (from 39.98 to 22.78 mN/m). When the pressure difference between both sides of the nylon membrane (Δ*P*) is higher than the substantial critical pressure for gas through the membrane (*P*_Critical (on)_), the transport gas will overcome the capillary force to open the liquid gate and the BPRLGM is open. Nevertheless, with UV light irradiation, the AzoC_8_F_15_ molecules convert to *cis*-form and its alignment density at the gating liquid surface decreases due to its increased steric hindrance, resulting in an increase in the surface tension of the gating liquid and a corresponding increase in substantial critical pressure for gas through the membrane (*P*_Critical (off)_). Thus, if the Δ*P* is lower than *P*_Critical (off)_, the BPRLGM is closed. Additionally, when the UV light is replaced by visible light again, the AzoC_8_F_15_ molecules with *cis*-form will rapidly reverse to *trans*-form, making the BPRLGM return to an open state. Based on the advantages of high spatiotemporal resolution and contactless control of photo stimuli, BPRLGM has potential applications in the precise and contactless control of microfluidics.

**Figure 2:**
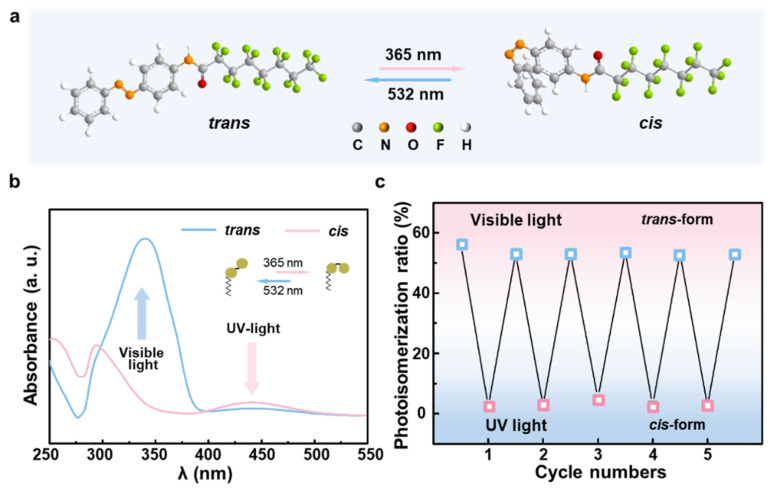
Design and evaluation of the photo-responsive surfactant molecule. (**a**) The photoisomerization of AzoC_8_F_15_ molecule under UV and visible light irradiations. (**b**) UV-vis absorption spectra of AzoC_8_F_15_ molecule with *trans* and *cis* forms. (**c**) Photoisomerization ratio of AzoC_8_F_15_ molecule during alternative irradiation cycles.

As a class of common molecular photoswitches, the azobenzene derivatives can be instantly triggered by UV and visible light to achieve reversible photochemical *trans*-to-*cis* isomerization [29]. Using a facile one-step substitution method, the AzoC_8_F_15_ molecules are obtained through combining a polar photo-responsive azobenzene group with a non-polar fluorocarbon chain, and the molecular polarity of the AzoC_8_F_15_ molecules will significantly change under different light irradiations [30]. As shown in Figure 2a, the *trans*-form of an AzoC_8_F_15_ molecule undergoes a conformational change to the *cis*-form under UV light irradiation (365 nm), and the *cis*-form of an AzoC_8_F_15_ molecule reverses to the *trans*-form by visible light irradiation (532 nm). UV-visible absorption spectra have successfully demonstrated the reversible photoisomerization between *trans* and *cis* forms of this photo-responsive surfactant molecule (Figure 2b). As can be observed, the photo-responsive AzoC_8_F_15_ molecule undergoes the expected *trans*-to-*cis* photoisomerization and *cis*-to-*trans* reversion through UV and visible light irradiations, wherein the peaks at around 345 nm and 435 nm could be assigned to the π-π* and n-π* adsorptions, respectively. Additionally, the AzoC_8_F_15_ molecule shows negligible attenuation of the photoisomerization ratio even under several alternating UV and visible light irradiation cycles, indicating its stable and reversible photochemical isomerization ability (Figure 2c). Under visible light irradiation (top), the AzoC_8_F_15_ molecule shows *trans*-form. Whereas under UV light irradiation (bottom), this AzoC_8_F_15_ molecule undergoes a *trans*-to-*cis* photoisomerization.

**Figure 3:**
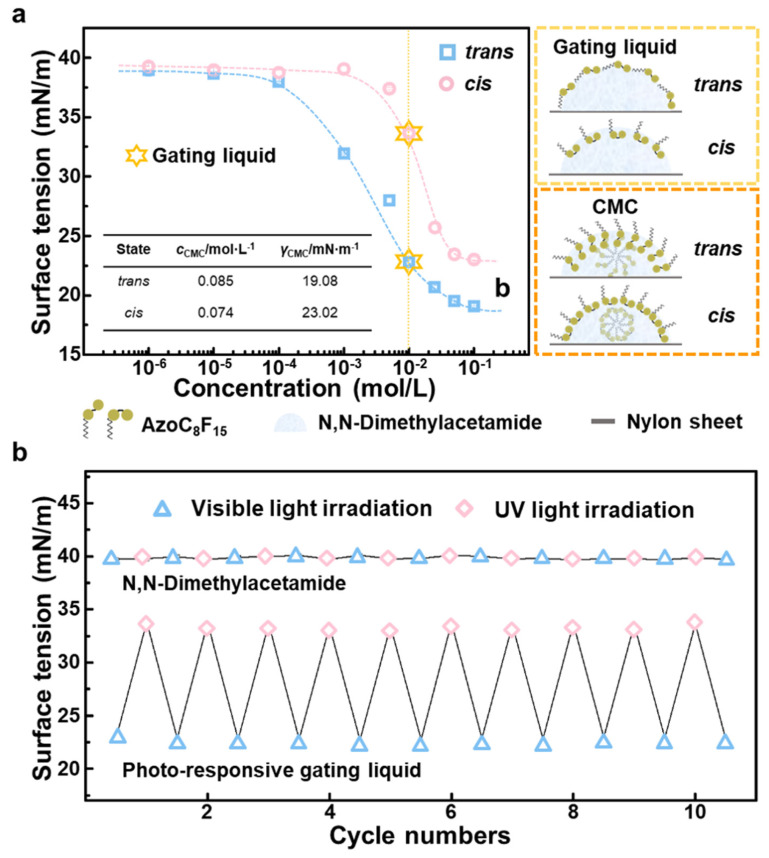
The selection of the photo-responsive gating liquid and its surface properties under UV and visible light irradiations. (**a**) Surface tension of AzoC_8_F_15_ solutions with different concentrations (left) and the alignments of AzoC_8_F_15_ molecules at the gas-liquid interface with concentrations of 0.01 mol/L and CMC (right). Blue squares and pink circles indicate the surface tension of AzoC_8_F_15_ molecules with *trans*- and *cis*-forms, respectively. Inset shows the critical micelle concentration (CMC) of AzoC_8_F_15_ molecules with *trans*- and *cis*-forms and the corresponding surface tension of its solution. (**b**) The surface tension of N,N-Dimethylacetamide (DMAC) and photo-responsive gating liquid during alternative irradiation cycles.

As mentioned above, the photoisomerization will change the molecular polarity of AzoC_8_F_15_ molecules, affecting the compatibility of surfactant molecules with the solvent. According to the Similarity–Intermiscibility Principle, the AzoC_8_F_15_ surfactant molecule with a non-polar fluorocarbon chain and a polar photo-responsive azobenzene group possesses weak compatibility with polar solvents, such as DMAC, which leads to the fast diffusion of surfactant molecules from the bulk phase to the gas-liquid interface. As a result, the diffusion of surfactant molecules induces a large reduction in the surface tension of the solution, and this ability to reduce the surface tension of a solution is called the surface activity of surfactant molecules [31]. Figure 3a shows the surface tensions of the photo-responsive surfactant solution with different concentrations of the AzoC_8_F_15_ molecules with *trans*- and *cis*-forms, reflecting the change in surface activity of AzoC_8_F_15_ molecules in DMAC solution under different light irradiations [32]. As can be observed, the surface tensions of AzoC_8_F_15_ solutions with both *trans*- and *cis*-form decreased obviously with an increase in the surfactant concentration, and eventually reached the plateau value around the critical micelle concentration (CMC) of surfactant [33]. As the concentration of the solution increased, due to the different alignments of the AzoC_8_F_15_ molecules with *trans*- and *cis*-forms at the gas-liquid interface, the surface tension of the surfactant solution shows significant variation under different light irradiations: when the surfactant concentration is 0.01 mol/L, the surface tensions of AzoC_8_F_15_ solutions with *trans*- and *cis*-forms are 22.79 and 33.63 mN/m, respectively, where the surface tension difference (Δγ = γ*_cis_*− γ*_trans_*) reached a maximum (Δγ*_max_* = 10.94 mN/m). This is because under photo stimuli, there is a noticeable change in the alignment of the AzoC_8_F_15_ molecule at the gas-liquid surface of the DMAC solution with 0.01 mol/L of AzoC_8_F_15_ molecules [34]. Under visible light irradiation, *trans*-formed AzoC_8_F_15_ molecules are non-polar and possess poor compatibility with the polar DMAC solvent, so it is easy for the molecules to diffuse to the gas-liquid surface of the solution. Following the Minimum Total Potential Energy Principle, *trans*-formed AzoC_8_F_15_ molecules tend to align horizontally at the gas-liquid surface, so that the gas-liquid surface is completely covered by non-polar molecules, and the surface tension of the solution is greatly reduced to 22.79 mN/m. However, the AzoC_8_F_15_ molecules undergo a *trans*-to-*cis* photoisomerization under UV light irradiation, resulting in an increase in the polarity of its azobenzene group. Thus, the interaction between the surfactant molecules and the polar DMAC solvent enhances, the azobenzene groups dissolve in DMAC solvent, and the non-polar fluorocarbon chains expose vertically to air. As a result, the solution surface tension increases to 33.63 mN/m due to the exposure of more DMAC solvent surface. According to the Laplace equation, the maximum surface tension difference corresponds to the maximum gas transmembrane critical pressure difference of the liquid gating membrane. Therefore, considering the practical application scenario of the liquid gating membrane, the DMAC solution of AzoC_8_F_15_ molecules with a concentration of 0.01 mol/L is selected as the photo-responsive gating liquid for the BPRLGM.

Under UV light irradiation, the AzoC_8_F_15_ molecules possess a *cis*-form. When the surfactant concentration of *cis*-formed AzoC_8_F_15_ molecules increased to 0.074 mol/L, all *cis*-formed AzoC_8_F_15_ molecules aligned vertically at the gas-liquid interface of the solution, exposing the nonpolar fluorocarbon chains to air. The surface tension of its solution achieved a minimum (23.02 mN/m), and this concentration is defined as the CMC of *cis*-formed AzoC_8_F_15_ molecules. Following this, the visible light irradiation was applied to the *cis*-AzoC_8_F_15_ solution, and the photo-responsive surfactant molecules underwent a *cis*-to*-trans* photoisomerization, which changed the alignment of the surfactant molecules at the gas-liquid interface of the solution. Differently to *cis*-formed AzoC_8_F_15_ molecules, *trans*-formed AzoC_8_F_15_ molecules closely align horizontally and completely cover the gas-liquid interface of the solution, resulting in a decrease in surface tension. Finally, we obtained the CMC of the *trans*-formed AzoC_8_F_15_ molecules of 0.085 mol/L, and the corresponding surface tension of its solution of 19.08 mN/m [35].

Furthermore, we investigated the surface tensions of the DMAC solvent and photo-responsive gating liquid under the alternating UV and visible light irradiations. As can be seen from Figure 3b, the surface tension of the DMAC remains almost constant during alternating irradiations with UV and visible light, indicating its stable and non-responsive surface property under photo-stimuli. On the contrary, the gating liquid (AzoC_8_F_15_ solution with a concentration of 0.01 mol/L) shows obvious and durable photo-responsive variation of surface tensions, which is mainly because of the reversible photoisomerization of AzoC_8_F_15_ molecules under alternating UV and visible light irradiations. Similarly, according to the Laplace equation, the reversible change in surface tension enables the substantial critical pressure for gas through the BPRLGM to be regulated by different photo-stimuli.

**Figure 4:**
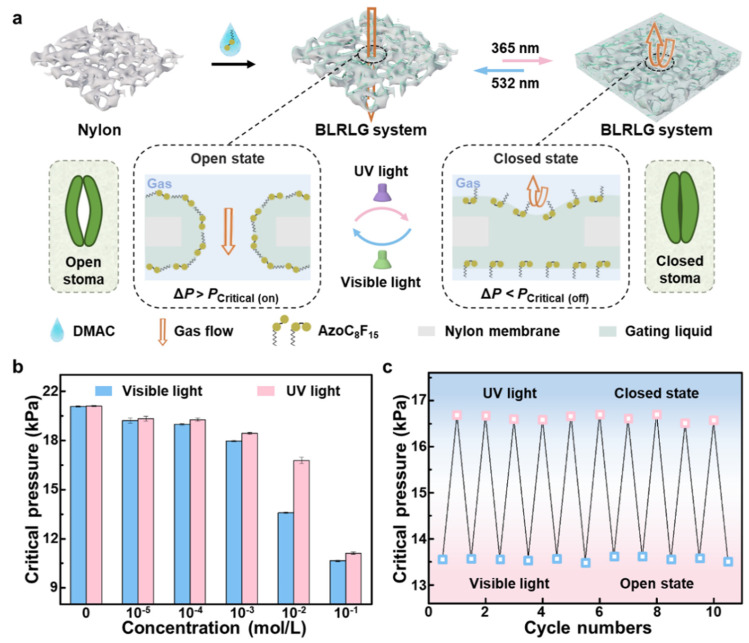
The photo-responsive gating performance of BPRLGM. (**a**) Interfacial design and preparation of BPRLGM. Illustration of the alignment of AzoC_8_F_15_ molecules at the gas-liquid interface of gating liquid during light-regulated gas transport and the corresponding stomatal behavior. (**b**) Critical pressures for gas through BPRLGM with different AzoC_8_F_15_ molecules concentrations under UV and visible light irradiations. (**c**) Cyclability of BPRLGM for gas under alternated UV and visible light irradiations.

The AzoC_8_F_15_ molecules are dissolved in the DMAC solvent as the photo-responsive gating liquid, and the nylon membrane works as a porous material to support the gating liquid. By simply infusing the gating liquid in the nylon membrane, the BPRLGM is established (Figure 4a). In this liquid gating membrane, the force required for the gas to overcome the capillary pressure at the gas-liquid interface is defined as the transmembrane critical pressure threshold, which is measured by a self-designed transmembrane pressure measurement device. The gating performance of the BPRLGM can be well explained by the Laplace equation. Under visible light irradiation, AzoC_8_F_15_ molecules with *trans*-form closely align horizontally at the gas-liquid interface, leading to a lower surface tension in the photo-responsive gating liquid, which means the substantial transmembrane critical pressure for gas through the BPRLGM (*P*_Critical (on)_) is relatively low. When a constant pressure difference (Δ*P*) higher than *P*_Critical (on)_ is applied on the BPRLGM, the transport gas slides open the liquid gate, creating an open liquid-lined pathway through the pores, and BPRLGM is open. This situation corresponds to the open state of the stoma in plant leaves under visible light irradiation. Through the open stoma, the effective gas transport involved in respiration, transpiration and photosynthesis on plant leaves can take place, and the life processes of the plants run normally. Whereas during UV light irradiation, *trans*-formed AzoC_8_F_15_ molecules undergo a *trans*-to-*cis* photoisomerization, and *cis*-formed AzoC_8_F_15_ molecules show lower alignment density than *trans*-form at the gas-liquid interface of the gating liquid due to their increased steric hindrance, resulting in an increase in the surface tension of the photo-responsive gating liquid [36]. A higher substantial critical pressure (*P*_Critical (off)_) is required for gas to pass through the BPRLGM, according to the Laplace equation. Furthermore, the gas cannot pass through the BPRLGM when the Δ*P* is lower than *P*_Critical (off)_ and the membrane is closed, which corresponds to the closed state of the stoma under UV light irradiation. For the closed stoma, water loss from plants will be avoided due to excessive transpiration. When visible light irradiation is applied to the membrane again, it returns to an open state. As a consequence, when a steady-state pressure Δ*P* (*P*_Critical (on)_ < Δ*P* < *P*_Critical (off)_) is applied to the BPRLGM, its closed and open states could be reversibly regulated by alternating UV and visible light irradiations [16].

Corresponding to the surface tensions of *trans*- and *cis*-formed AzoC_8_F_15_ solutions with different concentrations, we have studied the gas transmembrane critical pressure thresholds of the BPRLGM with different gating liquids under UV and visible light irradiations (Figure 4b). Compared with the DMAC liquid gating membrane, the critical pressures required for gas through the BPRLGM significantly decrease with the increase in gating liquid concentration under UV and visible light irradiations, and eventually reach a plateau value at the corresponding CMC, where the pressure thresholds are completely compliant with the Laplace equation [37,38]. When the concentration of the photo-responsive gating liquid is 0.01 mol/L, the BPRLGM shows the threshold variation of the substantial critical pressures of ~3.19 kPa under UV and visible light stimuli.

In addition, the BPRLGM with a gating liquid concentration of 0.01 mol/L exhibits switchable and stable photo-responsive gating performance for gas transport (Figure 4c). Under UV light irradiation (top), the AzoC_8_F_15_ molecules at the gas-liquid interface show *cis*-forms and higher surface tension of the gating liquid. When the applied constant pressure Δ*P* is lower than *P*_Critical (off)_, the BPRLGM is in the closed state. Whereas, under visible light irradiation (bottom), the AzoC_8_F_15_ molecules undergo a *cis*-to-*trans* photoisomerization, which leads to a decrease in the surface tension of the gating liquid. If Δ*P* is higher than the *P*_Critical (on)_, the gas will force the liquid gate and open the BPRLGM. After switching to UV light irradiation again, the AzoC_8_F_15_ molecules reverse to *cis*-forms, which will bring BPRLGM back to the closed state.

## 4. Conclusions

Inspired by the self-protection behavior of stomata under harsh light irradiation, we have designed a stable BPRLGM, imitating the photo-responsive behavior of the stomata in plants, which is established by infusing the azobenzene-based surfactant solution as the photo-responsive gating liquid into a nylon porous membrane. We evaluate the surface activities of the photo-responsive surfactant molecules, the photo-responsive properties of the gating liquid and the photo-responsive gating performance of the BPRLGM. The experimental results demonstrated that the AzoC_8_F_15_ molecules underwent the expected reversible *trans*-to-*cis* photoisomerization under alternating visible and UV light irradiations. Owing to the different alignments of *trans*- and *cis*-formed AzoC_8_F_15_ molecules at the gating liquid surface, the change in its surface tension reached its maximum at a concentration of 0.01 mol/L under UV and visible light irradiations, leading to the maximum substantial critical pressure difference for gas through the BPRLGM. Under alternating UV and visible light irradiations, the BPRLGM can realize reversible closed and open states driven by a pressure Δ*P*, which is consistent with the opening and closing of the stomata under external light stimuli. Consequently, the reversible switches on the open/closed states and the stability of the BPRLGM can be realized under alternating UV and visible light irradiations. Most the recent approaches are based on the variation of pore size to construct the bioinspired stomatal systems, which makes it difficult to achieve the real sense of smart gas transport. However, the BPRLGM can overcome this limitation well by utilizing liquid gating technology. In addition, the closing of the BPRLGM under UV light irradiation provides a new strategy for the design of smart materials that need to initiate self-protection behavior under harsh conditions, expanding applications in the industrial fields of bioinspired engineering materials, such as natural gas transport, multiphase separation and oil extraction.

## Data Availability

Not applicable.
